# Dairy Consumption and Incidence of Breast Cancer in the ‘Seguimiento Universidad de Navarra’ (SUN) Project

**DOI:** 10.3390/nu13020687

**Published:** 2021-02-21

**Authors:** Inmaculada Aguilera-Buenosvinos, Cesar Ignacio Fernandez-Lazaro, Andrea Romanos-Nanclares, Alfredo Gea, Rodrigo Sánchez-Bayona, Jose M. Martín-Moreno, Miguel Ángel Martínez-González, Estefanía Toledo

**Affiliations:** 1 Departamento de Medicina Preventiva y Salud Pública, Universidad de Navarra, 31008 Pamplona, Spain; iaguilera@alumni.unav.es (I.A.-B.); cflazaro@unav.es (C.I.F.-L.); aromanos@unav.es (A.R.-N.); ageas@unav.es (A.G.); mamartinez@unav.es (M.Á.M.-G.); 2 IdiSNA, Navarra Institute for Health Research, 31008 Pamplona, Spain; 3 Centro de Investigación Biomédica en Red Área de Fisiología de la Obesidad y la Nutrición (CIBEROBN), 28029 Madrid, Spain; 4 Department of Clinical Oncology, Hospital Universitario 12 de Octubre, 28041 Madrid, Spain; rodrosb@gmail.com; 5 Department of Preventive Medicine and Public Health, Medical School & INCLIVA, University of Valencia, 46010 Valencia, Spain; jose.martin-moreno@uv.es; 6 Department of Nutrition, Harvard T. H. Chan School of Public Health, Harvard University, Boston, MA 02115, USA

**Keywords:** breast cancer, dairy products, SUN cohort, Mediterranean population

## Abstract

Dairy products might influence breast cancer (BC) risk. However, evidence is inconsistent. We sought to examine the association between dairy product consumption—and their subtypes—and incident BC in a Mediterranean cohort. The SUN (“Seguimiento Universidad de Navarra”) Project is a Spanish dynamic ongoing cohort of university graduates. Dairy product consumption was estimated through a previously validated 136-item food frequency questionnaire (FFQ). Incident BC was reported in biennial follow-up questionnaires and confirmed with revision of medical records and consultation of the National Death Index. Hazard ratios (HR) and 95% confidence intervals (CI) were estimated with Cox regression models. Among 123,297 women-years of follow-up (10,930 women, median follow-up 12.1 years), we confirmed 119 incident BC cases. We found a nonlinear association between total dairy product consumption and BC incidence (*p*_nonlinear_ = 0.048) and a significant inverse association for women with moderate total dairy product consumption (HR_Q2vs.Q1_ = 0.49 (95% CI 0.28–0.84); HR_Q3vs.Q1_ = 0.49 (95% CI 0.29–0.84) *p*_trend_ = 0.623) and with moderate low-fat dairy product consumption (HR_Q2vs.Q1_ = 0.58 (95% CI 0.35–0.97); HR_Q3vs.Q1_ = 0.55 (95% CI 0.32–0.92), *p*_trend_ = 0.136). In stratified analyses, we found a significant inverse association between intermediate low-fat dairy product consumption and premenopausal BC and between medium total dairy product consumption and postmenopausal BC. Thus, dairy products, especially low-fat dairy products, may be considered within overall prudent dietary patterns.

## 1. Introduction

Breast cancer is the leading cancer in women worldwide. There were 2.08 million new cases in 2018, accounting for 24.2% of all new cases of cancer in women. It is the fifth leading cause of death from cancer in women [1]. In 2018, it was estimated that 627,000 women died due to breast cancer, which approximately corresponds to 15% of all cancer deaths among women [2]. Breast cancer risk has an inherited component (up to 10% of breast cancer in Western countries is due to genetic predisposition) [3] and several reproductive and lifestyle factors have been proven to augment breast cancer risk [4,5]. For example, birth weight is directly associated with breast cancer risk. Childhood, adolescent, and early adulthood body size is inversely related to breast cancer risk. On the other hand, body size from middle adulthood onward is directly associated with postmenopausal breast cancer development [4,6]. The latter has been suggested to decrease the risk of premenopausal breast cancer, especially among Africans and Caucasians but not Asian women [7]. It has also been suggested to increase the risk of triple-negative breast cancer [8]. Another lifestyle factor associated with the risk of breast cancer is diet. Nevertheless, evidence in this field is still inconclusive, and several dietary factors have been addressed as potential risk factors for or protective factors against breast cancer [4,9].

One dietary factor for which evidence is still inconclusive in terms of breast cancer prevention is dairy product consumption. These inconsistencies may be due to the fact that dairy products are a complex and varied food group, which can be further classified according to their fat content or to being fermented or not. Dairy products have been suggested to be beneficial for breast cancer prevention mainly due to their content of calcium, vitamin D, and conjugated linoleic acids (CLA) [10,11,12,13,14,15,16,17], whereas their saturated fat content has been proposed to directly impact breast cancer risk through increased insulin-like growth factor I (IGF-I) concentrations [18,19,20,21]. Furthermore, some dairy products are fermented products, and fermented dairy foods are functional foods, rich in nutrients and with probiotic content, which may be beneficial in the prevention of breast cancer [22]. When the associations between milk or dairy products as a food or food group and breast cancer development have been addressed, no consistent association has been observed for milk and dairy product consumption [23,24,25,26]. As such, several studies in a meta-analysis estimated an inverse association between dairy product consumption and the risk of breast cancer [12], although another cohort study in North America found that higher consumption of milk was associated with a greater risk of breast cancer, when adjusted for soy intake [27]. Additionally, low-/reduced-fat milk intake has been inversely associated with mammographic density [28].

A meta-analysis published in 2015 [12] concluded that dairy product consumption was inversely associated with breast cancer risk. However, most of the included studies, as well as those published thereafter on this topic [29,30] were conducted in US and Northern European countries. It is also worth mentioning that neither of these studies adjusted for a global dietary pattern, and dairy product consumption may be framed within different overall dietary patterns. Accordingly, the traditional Mediterranean diet has been suggested as potentially beneficial for breast cancer prevention [31,32], and dairy products, especially nonfermented whole-fat dairy products, have traditionally been considered contrary to what is recommend in the traditional Mediterranean diet pattern. Hence, the objective of this study was to examine the association between dairy consumption—and its different subtypes—and the incidence of premenopausal or postmenopausal breast cancer risk in a middle-age Mediterranean cohort, taking into consideration the global dietary pattern.

## 2. Materials and Methods

### 2.1. Study Sample

**Figure 1:**
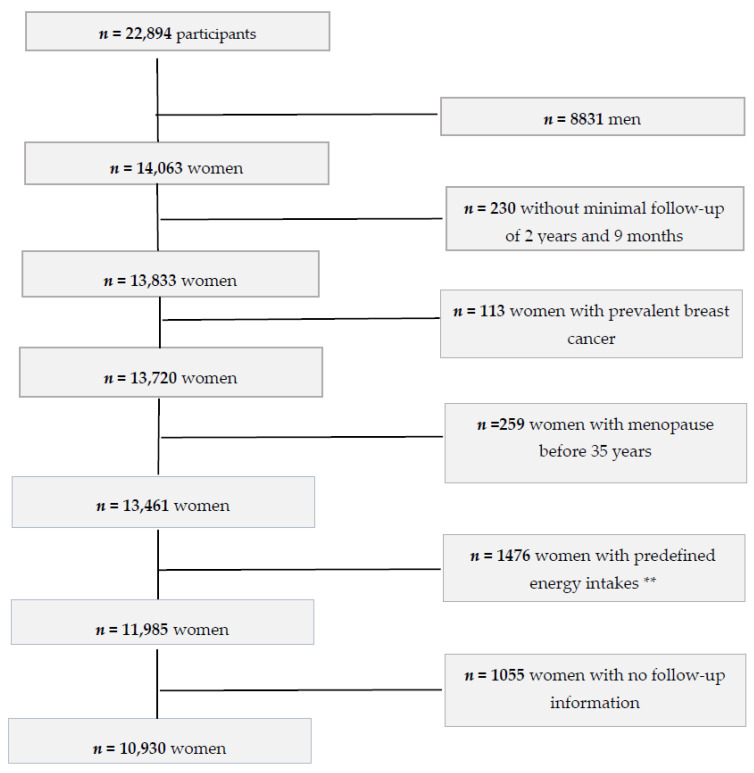
Flowchart representing the inclusion and exclusion criteria for the selection of participants of the SUN Project (“Seguimiento Universidad de Navarra”), included in this analysis. SUN Project, 1999–2019. * To ensure a 2 year and 9-months follow-up. ** Energy limits proposed by Willett (2013): 500–3500 kcal/day [34].

The “Seguimiento Universidad de Navarra” (SUN) (www.proyectosun.es, accessed on 20 January 2021) Project is an ongoing, multipurpose cohort of Spanish university graduates. The methodology was widely described elsewhere [33]. In brief, the SUN project is a dynamic cohort with a permanently open recruitment, aiming to investigate the association between diet and health-related outcomes. The methodology of the models of the Nurses’ Health Study and the Health Professionals Follow-Up Study served as an inspiration for the development of this cohort. Recruitment started in December 1999. Participants are middle-aged university graduates from different Spanish regions. Once the participants complete the first questionnaire, they become part of the cohort. Subsequently, follow-up questionnaires are sent biennially to update information which might have happened since they completed the previous questionnaire. For participants whose information is lost during the follow-up, the National Death Index is periodically consulted to confirm their vital status and, eventually, their cause of death. By the end of 2019, 22,894 participants were recruited (Figure 1). To guarantee a follow-up time of at least 2 years, we included only those participants who were recruited before March 2017. Out of 13,833 eligible women, we excluded 113 participants with self-reported history of breast cancer at baseline, 259 women who reported menopause before 35 years, 1476 women with implausible total energy intake (<500 or ≥3500 kcal/day) [34], and 1055 participants with no follow-up information (retention rate 91%). The final sample for the present study consisted of 10,930 participants who answered at least one follow-up questionnaire. The present study was in line with the guidelines stated in the Declaration of Helsinki, and all procedures involving participants were approved by the Institutional Review Board of the University of Navarra (30 August 2001). Voluntarily given informed consent through free fulfilment of the baseline questionnaire was gathered from all participants according to the methods approved by our Institutional Review Board.

### 2.2. Assessment of Dairy Consumption

Dairy products were assessed at baseline with a previously validated 136-item food frequency questionnaire (FFQ) [35]. The reproducibility of the FFQ was specifically assessed in this cohort [36]. Participants reported in the FFQ their habitual food consumption over the previous 12 months. It included 15 different dairy products and nine categories ranging from “never/seldom” to “more than six times per day”. We considered total dairy consumption, as well as whole-fat, low-fat, and fermented product consumption, as exposures. Table S1 (Supplementary Materials) describes the different dairy products which were considered in the different categories. To minimize within-person variation and reduce measurement error in exposures, we calculated the cumulative average of dairy product consumption by averaging repeated measures after 10 years of follow-up.

### 2.3. Breast Cancer Ascertainment

For the present analysis, the endpoint was incident breast cancer. During the follow-up, participants were inquired about any incident cases of breast cancer, and medical records were requested to confirm the diagnosis. Incident breast cancer cases were defined as women who developed breast cancer during the follow-up and were free of the disease at the beginning of the study (prevalent cases). They were also inquired about the date of diagnosis. Self-reported breast cancer cases were considered as probable cases. These participants were asked for a copy of their medical records. Then, a trained oncologist confirmed the cases on the basis of the medical records. These trained oncologists were blinded with respect to dietary exposures. Moreover, fatal breast cancer cases were reported to the research team by the subject’s next of kin, work associates, or postal authorities, along with the cause of death. For participants lost during the follow-up or with unidentified causes of death, the National Death Index was consulted. Finally, our analysis included 10,930 women among whom 119 developed breast cancer during the follow-up period (median follow-up = 12.1 years). 

### 2.4. Evaluation of Covariates 

At baseline, we also collected information about participants’ sociodemographic characteristics, medical history, health-related habits, lifestyles, anthropometric data, physical activity, and family history of breast cancer [37,38]. Age at menopause was updated in the questionnaire after 18 years of follow-up. For those women with no available information on age at menopause, we used the 75th percentile of the age of menopause (52 years in our sample) as the cut-off point [39]. Adherence to the Mediterranean diet was quantified on the basis of two scores: the score proposed by Trichopoulou et al. [34], which is based on the sample distribution for all items except of alcohol intake, and the 14-point Mediterranean Diet Adherence Screener (MEDAS) score [40,41,42], which is based on absolute consumptions. Both scores were modified to exclude the alcohol intake item, and the dairy product consumption item was further excluded from the score proposed by Trichopoulou et al. to avoid collinearity. The MEDAS score was included in a sensitivity analysis.

### 2.5. Statistical Analysis

For the main analysis, participants were divided into quartiles according to their reported consumption of total dairy, whole-fat, low-fat, and fermented product consumption. Baseline characteristics of participants were described as the means and standard deviations for quantitative traits or as percentages for qualitative ones according to quartiles of total dairy product consumption. Dairy product consumption and nutrient intakes were adjusted for total energy intake using the residual method [34].

To evaluate the association among baseline total, whole-fat, low-fat, and fermented dairy product consumption and the consequential risk of breast cancer, we used Cox regression models with age as the underlying time variable. Models were stratified for age (decades) and recruitment period. Comparisons had the lowest quartile of consumption as the reference category. We defined follow-up for each participant from the time of entry defined as the date of completion of the baseline questionnaire to the date of breast cancer diagnosis or breast cancer death for incident cases, or date of last follow-up or date of death for a cause other than breast cancer for non-cases. Probable incident cases were censored at the date of self-reported diagnosis. Hazard ratios (HR) and their 95% confidence interval (CI) were estimated. In an attempt to control for potential confounding factors, we used successive models of adjustment according to previous studies. Model 1 was adjusted for height, years at university, family history of breast cancer (none, after 45 years, or before 45 years), smoking status (never smoker, former smoker, current smoker), lifetime tobacco exposure (pack-years), physical activity (metabolic equivalents of task (METs)-h/week), television (TV)-watching (h/day), alcohol intake (g/day, continuous), body mass index (BMI) (<25, 25–30, ≥30 kg/m^2^), age of menarche (<10 years, 10–11 years, 12–13 years, ≥14 years), age at menopause (<50 years, ≥50 years), use of hormone replacement therapy (yes/no) and its duration (years) (continuous) only for postmenopausal women, history of pregnancy (age <25 years and nulliparous, age ≥25 years and nulliparous, first pregnancy before 25 years, first pregnancy between 25 and 30 years of age, first pregnancy being 30 years old or older), lifetime breastfeeding (months), energy intake (kcal/day), tertiles of calcium, vitamin D, and saturated fat from non-dairy products, intake of coffee consumption (<1, ≥1 cups/day), sugar-sweetened beverage consumption (never/seldom, ≥1 serving per week), and exposure to oral contraceptives (OC) (yes/no). The 2-year questionnaire included a nonspecific question about habitual medication use during the previous 2 years. Those participants who reported a habitual use of either oral combined contraceptives or oral progestin-only contraceptives were considered as OC users. We defined baseline exposure as having reported the habitual use of OC in the 2-year questionnaire, and model 2 was additionally adjusted for the Mediterranean diet adherence score proposed by Trichopoulou et al. [34] (without alcohol and dairy products items) (continuous). In postmenopausal women, we also adjusted for the time between recruitment and menopause. As sensitivity analysis, we adjusted for the MEDAS score instead of for the Mediterranean diet score proposed by Trichopoulou et al. (Table S1–S4 Supplementary Materials).

To evaluate dairy consumption during follow-up avoiding the effect of a variation in diet, Cox regression models were fitted with repeated dietary measurements using updated data from the FFQ after 10 years of follow-up (questionnaire C10). We used the cumulative average method for this analysis for those women with data information in the FFQ after 10 years. 

Subsequently, we stratified analysis according to menopausal status at breast cancer diagnosis. For the assessment of premenopausal breast cancer (*n* = 67) as the outcome, women who reported being menopausal before baseline assessment were excluded, and we censored follow-up time at the age of 52 years or at the self-reported age of menopause, whichever occurred first. When assessing postmenopausal breast cancer (*n* = 43), women were considered at risk only after having turned 52 years old or after their self-reported age of menopause, whichever occurred last. For the analyses with postmenopausal breast cancer as outcome, the multivariable models were additionally adjusted for time since recruitment until the beginning of the time at risk. This latter figure was 0 for women who were already postmenopausal at baseline. For initially premenopausal women who turned postmenopausal during follow-up, we calculated time since recruitment as the difference between the self-reported date of menopause and the date of completion of the baseline assessment. 

We also conducted tests of linear trend for the evaluation of dose–response relationships, assigning to each category of the total diary intake its quartile-specific median and using the resulting variable as continuous in the abovementioned models. Furthermore, to deeply understand the relationship between each exposure and the main outcome, flexible regression models (cubic splines) were used. Each model was controlled for all the covariates abovementioned. 

Assuming an alpha error of 0.05, an overall incidence of 1.1%, a Cox regression coefficient of −0.69—equivalent to an HR of 0.5—and a standard deviation of 0.5, our analyses would have a statistical power of 83%.

We also calculated the main sources of total dairy products and calcium from non-dairy products, as well as the main sources of variability in total dairy products in this Mediterranean cohort for both individual foods adjusted for the residual method. To calculate the contribution of each food item (or group of them) to the between-person variability dairy product consumption, we conducted nested regression analyses after a stepwise selection algorithm. The contribution of each food group is shown in the cumulative *R*^2^ change. Furthermore, we estimated their contribution related to total dairy product as the grams consumed from each food group divided by the total dairy products (%). 

Analyses were performed using STATA/SE version 12.0 (College Station, TX, USA: StataCorp LLC); we used two-sided *p*-values and the statistical significance threshold was set a priori at 0.05.

## 3. Results

The main baseline characteristics of the 10,930 women included in our analyses according to the quartiles of baseline total dairy product consumption are shown in Table 1. The mean age of participants was 35 years (standard deviation (SD) 10.6), and the median BMI was 22.4 kg/m^2^ (SD 10.7). Women in the highest category of total dairy product consumption were more physically active and more likely to be never smokers. They also consumed less alcohol per day but more coffee per day than women with lower dairy product consumption. 

**Figure 2:**
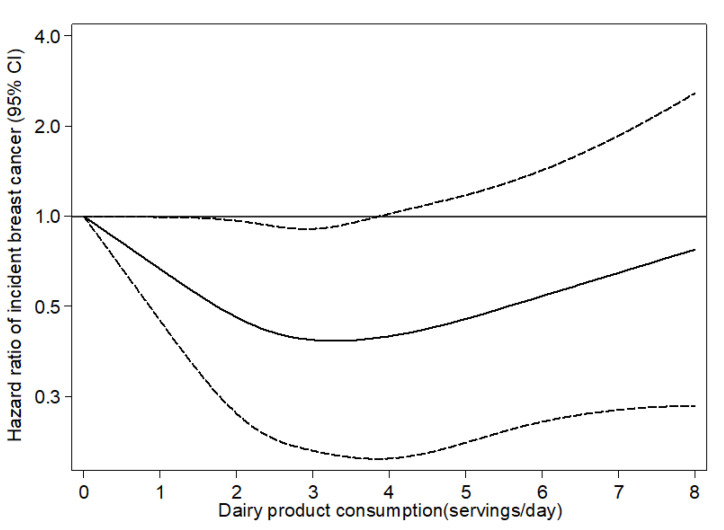
Restricted cubic splines for the hazard ratio (HR) and 95% confidence intervals (CIs) of total intake of total dairy product consumption incidence of breast cancer in the “Seguimiento Universidad de Navarra” (SUN) cohort. The black line represents the HR, and the dashed lines represent the 95% CIs. A serving/day value of 0 was the reference value. Adjusted for height, years at university, family history of breast cancer (none, after 45 years, or before 45 years), smoking status (never smoker, former smoker, current smoker), lifetime tobacco exposure (pack-years), physical activity (METs-h/week), TV watching (h/day), alcohol intake (g/day, continuous), BMI (<25, 25–30, ≥30), age of menarche (<10 years, 10–11 years, 12–13 years, ≥14 years), age at menopause (<50 years, ≥50 years), history of pregnancy (age <25 years and nulliparous, age ≥25 years and nulliparous, first pregnancy before 25 years, first pregnancy between 25 and 30 years of age, first pregnancy being 30 years old or older), months of breastfeeding (continuous), use of hormone replacement therapy (yes/no) and its duration (continuous), energy intake (kcal/day), energy-adjusted intake of calcium, vitamin D, and saturated fat from non-dairy products (tertiles), coffee consumption (<1, ≥1), sugar-sweetened beverage consumption (never/seldom, ≥1 serving per week), and oral contraceptives (yes/no), as well as additionally adjusted for Mediterranean Diet Adherence (score Trichopoulou without alcohol and dairy product items) (continuous); *p* for nonlinearity = 0.048.

We confirmed 119 new-onset cases of breast cancer among 123,297 person-years of follow-up (median follow-up 12.1 years). We found a significant nonlinear association between total dairy product consumption and the incidence of breast cancer (*p* for nonlinearity = 0.048; Figure 2). Concretely, we observed a U-shaped association such that the lowest risk was observed among women with a dairy product consumption of 2–4 servings/day. In fact, we observed a significant inverse association for women in the second and third quartile of total dairy product consumption—with median consumption of 2–4 servings per day of total dairy products—compared to women in the first quartile (adjusted HR_Q2 vs. Q1_ 0.49; 95% CI 0.28–0.84; *p* = 0.009 and HR_Q3 vs. Q1_ 0.49; 95% CI 0.29–0.84; *p* = 0.009) (Table 2). These associations barely changed when we adjusted for the adherence to the Mediterranean diet with the MEDAS score instead of the Mediterranean diet score proposed by Trichopoulou et al. (Table S2, Supplementary Materials). We also observed inverse associations between low-fat dairy consumption and overall breast cancer for women with intermediate low-fat dairy product consumption compared to women in the lowest category of low-fat dairy consumption (adjusted HR_Q2 vs. Q1_ 0.58; 95% CI 0.35–0.97; *p* = 0.039 and HR_Q3 vs. Q1_ 0.55; 95% CI 0.33–0.92; *p* = 0.023). We observed no significant associations when we addressed the consumption of whole-fat or fermented dairy product consumption and total breast cancer. No significant interactions with adherence to the Mediterranean diet were observed.

When we stratified participants according to menopausal status, we found no significant association between total dairy product consumption and incidence of premenopausal breast cancer, but we observed a significant inverse association between intermediate consumption of low-fat dairy products and premenopausal breast cancer risk (*n* = 67) (adjusted HR_Q2 vs. Q1_ 0.26; 95% CI 0–0.59; *p* = 0.001 and adjusted HR_Q3 vs. Q1_ 0.48; 95% CI 0.25–0.92; *p* = 0.027) (Table 3). Results barely changed when we used the 14-point MEDAS score to assess adherence to the Mediterranean diet (Table S3, Supplementary Materials). Non significant associations were observed for whole-fat and fermented dairy product consumption. 

On the other hand, postmenopausal women who showed a medium total dairy product consumption (2–4 servings/day) showed a significant inverse association with postmenopausal breast cancer (adjusted HR_Q2 vs. Q1_ 0.28; 95% CI 0.10–0.76; *p* = 0.012 and adjusted HR_Q3 vs. Q1_ 0.42; 95% CI 0.18–0.96; *p* = 0.040) (Table 4). Results were consistent when we considered the MEDAS score to adjust for adherence to the Mediterranean diet (Table S4, Supplementary Materials). No significant associations were found considering subtypes of dairy product consumption.

Skimmed milk contributed the most to the between-person variability when we adjusted for the residual method. On the other hand, whole-fat yogurt contributed the most to the between-person variability without adjusting for the residual method. In terms of quantity, semi-skimmed and skimmed milk contributed most to the total dairy product consumption.

## 4. Discussion

We found in this Mediterranean cohort that a moderate consumption of total dairy products (2–4 servings/day) was inversely associated with overall and postmenopausal incidence of breast cancer. Moreover, we found that moderate consumption of low-fat dairy products (1–2 servings/day) was significantly and inversely associated with total and premenopausal breast cancer. No significant associations were observed for whole-fat or fermented dairy products and breast cancer risk. 

### 4.1. Evidence from Previous Epidemiological Studies 

The observed inverse association between all dairy products and overall breast cancer was generally consistent with previous studies. In a meta-analysis including 22 prospective studies, modest and high consumption of total dairy products were associated with a 6% reduced risk of breast cancer [12]. The associations were somewhat stronger for low-fat dairy products than for high-fat dairy products and for premenopausal women. It is worth mentioning that, in that meta-analysis, they observed a linear inverse association between dairy product consumption and breast cancer risk, and we found a U-shape association wherein consumption ranges between two and four servings showed an inverse association with breast cancer risk, which is comparable to the inverse linear trend observed in the abovementioned meta-analysis. Two further prospective studies were published after that meta-analysis [29,30]. Farvid et al. carried out a prospective cohort study on the relationship between early adulthood dairy consumption and breast cancer in the Nurses’ Health Study II cohort. Additionally, Kaluza et al. examined whether long-term consumption of milk and fermented dairy products was associated with the risk of breast cancer in the Swedish Mammography Cohort, composed mainly of postmenopausal women; nevertheless, they did not consider the association for total or whole-fat and low-fat dairy products. Out of the aforementioned studies, only the European Prospective Investigation into Cancer and Nutrition (EPIC) study and the French Supplementation en Vitamines et Mineraux Antioxydants (SU.VI.MAX) study included participants from Mediterranean countries [43,44]. Nevertheless, results from EPIC were not stratified by country or region, and the dietary patterns within dairy product consumption may be different in different countries. Results from the French SU.VI.MAX cohort study [44] found a lower risk of breast cancer with high total dairy product consumption in the whole population, which is consistent with our results obtained for overall breast cancer. Nevertheless, in the SU.VI.MAX study, dairy product consumption was studied as a continuous variable, and the number of incident cases did not allow for stratification by menopausal status. In addition, none of the cited studies [12,29,30] adjusted for the overall dietary pattern. Thus, there may be some residual confounding by overall dietary pattern when addressing the association between dairy products and breast cancer. However, we found no significant interaction with the adherence to the Mediterranean diet such that our results did not seem to change with a different overall dietary pattern. Higher consumption of dairy products was associated with circulating steroid hormone concentrations in postmenopausal women in the Melbourne Collaborative Cohort Study (MCCS) [45]. When we separately carried out our analyses by menopausal status, we found a statistically significant inverse association between dairy product consumption and postmenopausal breast cancer. This finding is consistent with the Cancer Prevention Study II Nutrition Cohort [46], which concluded that the consumption of at least two servings of dairy products per day was inversely associated with the risk of breast cancer among postmenopausal women. It is important to highlight that the range of dairy product consumption in which they found an inverse relationship with breast cancer was similar to ours. However, results from the Rotterdam Study [23] observed no consistent association between dairy products and breast cancer among women aged 55 years or older. Nevertheless, the combined estimate for the association between total diary consumption and specifically postmenopausal breast cancer did not reach statistical significance in the latest available meta-analysis (relative risk (RR) 0.94 (95% CI: 0.86–1.02) for high vs. low dairy product consumption) [12]. Despite us finding no significant associations between total dairy consumption and the risk of premenopausal breast cancer, it is noteworthy that the confidence intervals widely overlapped with the results for overall breast cancer incidence, and limited statistical power may be an alternative explanation for our null findings in this subgroup.

Dairy products are a heterogeneous food group which can be further classified according to their fat content or their fermentation. As for the first distinction, low-fat dairy products differ from high-fat dairy products in terms of the fatty acid content, and they are produced by filtering full-fat dairy to remove most saturated fatty acids, while maintaining unsaturated fatty acids [10]. In the previously mentioned meta-analysis by Zhang et al. [12], a stronger inverse association for low-fat dairy product consumption than for total dairy product consumption was observed. In our study, despite the point estimates for modest low-fat dairy product consumption being <1 for overall and postmenopausal breast cancer, the association yielded statistical significance only for premenopausal breast cancer. 

An interesting aspect to consider is the timing of consumption, which might be important in terms of breast cancer prevention. In subgroup analyses, Zhang et al. observed an inverse association between total dairy product intake and breast cancer incidence only for premenopausal breast cancer but not for postmenopausal breast cancer or among studies which addressed childhood consumption and subsequent breast cancer risk [12]. In line with those results, Farvid et al. [29] examined dairy consumption during early life (adolescence and early adulthood) in relation to incident breast cancer in the Nurses´ Health Study II Cohort. They concluded that no overall association was found. Although they did not find a significant association between early adulthood high-fat dairy foods and overall breast cancer, a significant direct association was identified among women younger than 45 years, underlining the importance of appropriately addressing the susceptibility window for dairy products to influence breast cancer risk.

### 4.2. Biological Mechanisms 

Regarding biological mechanisms, dairy products contain components which have been proposed to be both potentially beneficial and potentially detrimental in terms of breast cancer prevention. On the one hand, dairy products, especially whole-fat dairy products, are a source of high saturated fat, which is widely associated with breast cancer risk [47,48]. On the other hand, dairy products and especially milk are a dietary source of IGF-1, and evidence from laboratory studies suggests that overexpression of IGF-1 is involved in cancer pathogenesis through mitogenic and antiapoptotic effects [21,49,50]. However, other minor components of dairy products as calcium [51], vitamin D [52], andCLA [53] have all been suggested to reduce breast cancer risk though several mechanisms due to their participation in controlling cell growth in normal and malignant breast cells. One further mechanism through which dairy products may help prevent breast cancer is through the modulation of insulin resistance. In fact, high consumption of skimmed dairy has been associated with a lower risk of altered glucose metabolism [54,55], as well as activation of the IGF-1 signaling among others [56]. Higher insulin resistance and IGF-1 concentrations have, in turn, also been linked to a higher risk of breast cancer [57,58]. Lastly, a direct association between dairy product consumption and circulating steroid hormone concentrations has been observed [45]. Higher concentrations of circulating steroid hormone concentrations have been related to a higher risk of breast cancer and, more specifically, to postmenopausal breast cancer [59].

### 4.3. Limitations and Strengths

Our study has various potential limitations to take into consideration. First, our statistical power could be limited, because of the observed number of incident cases of breast cancer, especially when considering postmenopausal breast cancer. We acknowledge that, due to the multiplicity of the analysis and comparisons considered, results must be interpreted cautiously. Our cohort mostly represent young Mediterranean women, among whom the incidence of breast cancer is lower. Second, information on breast cancer incidence was self-reported. We are aware that we might have lost some breast cancer cases. Nevertheless, the identified age-adjusted incidence was consistent with the reported incidence of breast cancer in the Spanish population [60]. In addition, in order to avoid false positives, breast cancer cases were confirmed by a blinded trained oncologist as explained earlier. Third, self-reported information of exposure could denote some degree of misclassification which, in turn, may have biased our results toward the null. However, dietary information was assessed with a previously validated semi-quantitative FFQ [35]. The FFQ is able to assess dietary intakes and complex information over a longer period of time, which is exactly its function within the SUN prospective cohort. Other advantages to be highlighted is that the FFQ is representative of “habitual” intake, as well as being significantly less expensive and suitable for very large studies. On the other hand, it is a retrospective method than relies upon the memory of responders, and it is less sensitive to measures of absolute intake of specific nutrients [61]. In this regard, intraclass correlation coefficient with repeated 3-day dietary records for dairy products was 0.84 in the validation study. Fourth, we did not have a representative sample of the general population. However, lack of representativeness does not preclude from addressing measures of association. Fifth, the fact that there is no standard method available to categorize dairy product intake leads many studies like ours to base their analysis on the categorization of quartiles or quintiles within a specific study population. In spite of that, our study also has some strengths. It has a longitudinal design which reduces the possibility of reverse causation bias. We adjusted our analyses for a wide range of potential confounders, although we cannot completely rule out the possibility of residual confounding. Moreover, the adjustment for potential confounders and the sensitivity analyses reinforce our findings. Self-reported cancer cases were confirmed through medical reports to guarantee that the final diagnosis was an invasive breast carcinoma. We assessed the robustness of the results using two different scores for assessment of Mediterranean Diet adherence. Lastly, we used repeated measurements of dietary information to reduce the effect of variation in diet.

## 5. Conclusions

Our results suggest an inverse association between intermediate total dairy product consumption and overall and postmenopausal breast cancer and between intermediate low-fat dairy product consumption and total and premenopausal breast cancer. Thus, dairy products, especially low-fat dairy products, may be incorporated within overall prudent dietary patterns. Future insight into the role of dairy product consumption in breast cancer may be obtained by separately assessing the associations of high- and low-fat dairy products. 

## Figures and Tables

**Table 1:** Baseline characteristics of 10,930 women in the SUN cohort according to the baseline total dairy product consumption.

	Quartiles of Energy Adjusted Dairy Product Consumption			
	Q1	Q2	Q3	Q4
*N*	2733	2732	2733	2732
Median energy-adjusted dairy product consumption (servings/day)	1.5	2.5	3.3	4.7
Median energy-adjusted dairy product consumption range (servings/day)	≤2	2–3	3–4	≥4
Median energy-adjusted whole-fat dairy product consumption (servings/day)	0.4	1.0	1.6	2.8
Median energy-adjusted low-fat dairy product consumption (servings/day)	0.7	0.9	1.7	3.2
Median energy-adjusted fermented dairy product consumption (servings/day)	0.5	1.0	1.5	2.6
Median total milk consumption (servings/day)	0.7	1	1.3	2.5
Median total yogurt consumption (servings/day)	0.1	0.4	0.6	1
Median total cheese consumption (servings/day)	0.2	0.5	0.6	0.9
Time of university education (years), mean (SD)	4.8 (1.3)	4.9 (1.3)	4.9 (1.3)	4.8 (1.4)
Age (years), mean (SD)	35.6 (10.8)	34.9 (10.5)	35.0 (10.3)	35.4 (10.7)
Height (cm), mean (SD)	163.6 (6.1)	163.7 (6.0)	163.5 (6.0)	163.6 (6.1)
Body max index (kg/m^2^), mean (SD)	22.0 (3.1)	22. 2 (3.1)	22.3 (3.0)	22.4 (3.0)
Physical activity (METs-h/week), mean (SD)	17.7 (19.6)	17.8 (18.5)	19.2 (19.3)	20.4 (21.1)
Total energy intake (kcal/day), mean (SD)	2392 (588)	2221 (561)	2256 (562)	2314 (566)
Alcohol intake (g/day), mean (SD)	4.9 (7.0)	4.2 (5.8)	3.7 (5.4)	3.3 (5.1)
Hours/day television watching, mean (SD)	1.7 (1.3)	1.6 (1.2)	1.6 (1.2)	1.6 (1.2)
Sugar-sweetened beverage consumption (%)				
Never or seldom	58.5	60.2	63.3	68.3
≥1 servings/week	41.5	39.8	36.7	31.6
Age at menarche (%)				
Early	1.1	1.0	1.1	1.3
10–11 years	17.6	19.8	18.7	20.5
12–13 years	55.7	54.9	55.3	53.5
≥14 years	25.5	25.1	24.8	24.6
Obstetric history (%)				
Age <25 years and nulliparous	18.0	18.1	17.4	17.5
Age ≥25 years and nulliparous	49.3	50.7	48.6	47.6
First pregnancy before 25 years	4.5	4.1	4.8	5.1
First pregnancy between 25 and 30 years of age	14.1	13.6	15.2	14.7
First pregnancy being 30 years old or older	13.9	13.2	13.8	14.9
Oral contraceptive use (%)				
No	97.6	97.1	97.7	97.7
Yes	2.3	2.8	2.3	2.3
Menopausal status at recruitment (%)				
Premenopausal (%)	91.9	92.6	92.6	91.5
Postmenopausal (%)	8.0	7.4	7.4	8.5
Age at menopause (%) ^a^				
<50	4.1	3.8	4.1	4.5
≥50	3.9	3.6	3.3	3.9
Family history of breast cancer (%) ^b^				
None	89.3	89.9	89.3	88.9
Before 45 years	1.7	1.7	2.0	1.8
After 45 years	8.9	8.4	8.6	9.3
Hormone replacement therapy (%) ^c^				
No	95.9	95.9	95.2	94.1
Yes	4.1	4.1	4.8	5.9
Time of hormone replacement therapy (years) mean (SD) ^c^	0.1 (0.9)	0.1 (0.8)	0.1 (0.8)	0.2 (1.1)
Breastfeeding (months), mean (SD)	2.3 (4.9)	2.2 (4.7)	2.4 (5.0)	2.4 (5.1)
Smoking status (%)				
Never	47.8	52.4	54.3	52.1
Current	26.5	22.7	20.7	20.8
Former	25.7	25.9	24.9	27.1
Lifetime tobacco exposure (pack-years), mean (SD)	4.7 (7.5)	4.0 (6.9)	3.9 (6.9)	4.3 (7.4)
Energy-adjusted calcium intake from non-dairy product (mg/day), mean (SD)	1514 (1774.8)	1383 (1428.6)	1394 (1328.2)	1409(1583.4)
Energy-adjusted vitamin D intake from non-dairy product (mg/day), mean (SD)	6.1(5.1)	6 (4)	6 (4.2)	6 (4.1)
Energy-adjusted saturated fat intake from non-dairy product (mg/day), mean (SD)	23.8 (6.4)	22.4 (5.2)	21.3 (5)	18.9 (5.2)
Coffee consumption (servings/day), (%)				
<1	39.7	36.0	34.0	31.6
≥1	60.3	63.9	65.9	68.3
Adherence to Mediterranean diet, mean (SD) ^d^	3.5 (1.6)	3.3 (1.6)	3.4 (1.6)	3.5 (1.6)

Values are expressed as the mean (SD) for quantitative variables and as percentage for categorical ones. Medians are expressed for quartiles of exposure variables (servings/day). Interquartile ranges are expressed for the group variable. MET: metabolic equivalent of task. ^a^ For women with no available information on age at menopause, we used the 75th percentile of the age of menopause (52 years in our sample). ^b^ Information from mother, sisters, and both grandmothers was collected. ^c^ Only for postmenopausal women. ^d^ Score proposed by Trichopoulou et al. (2013) [34].

**Table 2:** Hazard ratio (HR) and 95% confidence intervals (CI) of confirmed breast cancer cases according to the categories of baseline total dairy, whole-fat, low-fat, and fermented dairy product consumption among 10,930 women of the SUN Project.

	**Total Dairy Product Consumption**				
	**Q1 (Ref.)**	**Q2**	**Q3**	**Q4**	***p* for trend**
*N*	2733	2732	2733	2732	
Median total dairy product consumption	1.6	2.5	3.4	4.8	
Incident cases	41	23	19	36	
Person-years of follow-up	30,609	30,506	31,368	30,811	
Incidence rate/10,000 person-years	13.39	7.53	6.05	11.68	
Age-adjusted HR (95% CI)	1.00 (Ref.)	0.59 (0.35–0.99)	0.45 (0.26–0.78)	0.89(0.57–1.39)	0.665
Multivariable adjusted model 1	1.00 (Ref.)	0.64 (0.37–1.09)	0.53 (0.28–1.00)	1.19 (0.61–2.31)	0.496
Multivariable adjusted model 2	1.00 (Ref.)	0.63 (0.37–1.09)	0.52 (0.28–0.97) *	1.17 (0.60–2.30)	0.523
Repeated measurements	1.00 (Ref.)	0.49 (0.28–0.84) **	0.49 (0.29–0.84) **	0.84 (0.52–1.35)	0.623
	**Whole-Fat Dairy Product Consumption**				
	**Q1 (Ref.)**	**Q2**	**Q3**	**Q4**	***p* for trend**
*N*	2733	2732	2733	2732	
Median whole-fat dairy product consumption	0.6	1.19	1.18	3.1	
Incident cases	29	32	28	30	
Person-years of follow-up	29,397	29,672	31,367	32,859	
Incidence rate/10,000 person-years	9.86	10.78	8.92	9.12	
Age-adjusted HR (95% CI)	1.00 (Ref.)	1.19 (0.71–1.97)	1.01 (0.60–1.72)	1.09 (0.65–1.83)	0.886
Multivariable adjusted model 1	1.00 (Ref.)	1.27 (0.75–2.15)	1.08 (0.62–1.88)	1.11 (0.63–1.95)	0.912
Multivariable adjusted model 2	1.00 (Ref.)	1.27 (0.75–2.15)	1.07 (0.61–1.88)	1.10 (0.66–1.95)	0.915
Repeated measurements	1.00 (Ref.)	1.27 (0.76–2.14)	1.09 (0.63–1.87)	1.05 (0.61–1.79)	0.830
	**Low-Fat Dairy Product Consumption**				
	**Q1 (Ref.)**	**Q2**	**Q3**	**Q4**	***p* for trend**
*N*	2,733	2732	2,733	2732	
Median low-fat dairy product consumption	0.67	0.68	1.5	3	
Incident cases	44	23	24	28	
Person-years of follow-up	33,043	30,357	29,727	29,727	
Incidence rate/10,000 person-years	13.31	7.57	8.07	9.28	
Age-adjusted HR (95% CI)	1.00 (Ref.)	0.57 (0.34–0.95)	0.58 (0.35–0.96)	0.63 (0.39–1.03)	0.113
Multivariable adjusted model 1	1.00 (Ref.)	0.65 (0.39–1.08)	0.69 (0.40–1.17)	0.80 (0.44–1.44)	0.440
Multivariable adjusted model 2	1.00 (Ref.)	0.65 (0.39–1.09)	0.69 (0.40–1.17)	0.79 (0.44–1.44)	0.422
Repeated measurements	1.00 (Ref.)	0.58 (0.35–0.95) *	0.55 (0.32–0.92) *	0.65 (0.39–1.06)	0.136
	**Fermented Dairy Product Consumption**				
	**Q1 (Ref.)**	**Q2**	**Q3**	**Q4**	***p* for trend**
*N*	2733	2732	2733	2732	
Median fermented dairy product consumption	0.5	1.04	1.6	2.6	
Incident cases	30	29	33	27	
Person-years of follow-up	31,229	30,973	30,896	30,196	
Incidence rate/10,000 person-years	9.60	9.36	10.68	8.94	
Age-adjusted HR (95% CI)	1.00 (Ref.)	0.99 (0.59–1.65)	1.08 (0.66–1.78)	0.91 (0.54–1.53)	0.746
Multivariable adjusted model 1	1.00 (Ref.)	1.16 (0.68–1.96)	1.32 (0.77–2.24)	1.23 (0.68–2.23)	0.522
Multivariable adjusted model 2	1.00 (Ref.)	1.16 (0.68–1.96)	1.32 (0.77–2.24)	1.23 (0.68–2.23)	0.515
Repeated measurements	1.00 (Ref.)	1.09 (0.65–1.85)	1.06 (0.63–1.79)	0.99 (0.57–1.72)	0.841

* *p* < 0.05, ** *p* < 0.01. Ref., reference. Results from Cox regression models. All Cox models were stratified for age (decades) and recruitment period. Model 1 additionally adjusted for height, years at university, family history of breast cancer (none, after 45 years, or before 45 years), smoking status (never smoker, former smoker, current smoker), lifetime tobacco exposure (pack-years), physical activity (METs-h/week), TV watching (h/day), alcohol intake (g/day, continuous), BMI (<25, 25–30, ≥30), age of menarche (<10 years, 10–11 years, 12–13 years, ≥14 years), age at menopause (<50 years, ≥50 years), history of pregnancy (age <25 years and nulliparous, age ≥25 years and nulliparous, first pregnancy before 25 years, first pregnancy between 25 and 30 years of age, first pregnancy being 30 years old or older), months of breastfeeding (continuous), use of hormone replacement therapy (yes/no) and its duration (continuous), energy intake (kcal/day), energy-adjusted intake of calcium, vitamin D, and saturated fat from non-dairy products (tertiles), coffee consumption (<1, ≥1), sugar-sweetened beverage consumption (never/seldom, ≥1 serving per week), and oral contraceptives (yes/no). Model 2 additionally adjusted for Mediterranean diet adherence (score Trichopolou) (continuous). Repeated measurements were adjusted for the same variables as Model 1, using cumulative averages for all dietary variables.

**Table 3:** Hazard ratio (HR) and 95% confidence intervals (CI) of confirmed premenopausal breast cancer cases according to the categories of baseline total dairy, whole-fat, low-fat, and fermented dairy product consumption among 9971 women of the SUN Project.

	**Total Dairy Product Consumption**				
	**Q1 (Ref.)**	**Q2**	**Q3**	**Q4**	***p* for trend**
*N*	2493	2493	2493	2492	
Mean whole-fat dairy product consumption	1.8	2.7	3.6	4.9	
Incident cases	20	16	9	22	
Person-years of follow-up	23,954	24,344	25,245	24,389	
Incidence rate/10,000 person-years	8.34	6.57	3.56	9.02	
Age-adjusted HR (95% CI)	1.00 (Ref.)	0.84 (0.43–1.62)	0.42 (0.19–0.92)	1.08 (0.58–1.98)	0.933
Multivariable adjusted model 1	1.00 (Ref.)	0.88 (0.43–1.77)	0.50 (0.20–1.27)	1.37 (0.53–3.51)	0.447
Multivariable adjusted model 2	1.00 (Ref.)	0.88 (0.43–1.78)	0.51 (0.20–1.28)	1.36 (0.53–3.53)	0.456
Repeated measurements	1.00 (Ref.)	0.75 (0.37–1.50)	0.51 (0.24–1.10)	1.00 (0.52–1.91)	0.991
	**Whole-Fat Dairy Product Consumption**				
	**Q1 (Ref.)**	**Q2**	**Q3**	**Q4**	***p* for trend**
*N*	2493	2493	2493	2492	
Mean whole-fat dairy product consumption	0.6	1.2	1.8	3	
Incident cases	16	14	17	20	
Person-years of follow-up	21,544	23,533	25,522	27,332	
Incidence rate/10,000 person-years	7.42	5.94	6.66	7.31	
Age-adjusted HR (95% CI)	1.00 (Ref.)	0.76 (0.37–1.57)	0.94 (0.47–1.85)	1.08 (0.56–2.08)	0.603
Multivariable adjusted model 1	1.00 (Ref.)	0.82 (0.39–1.73)	0.94 (0.45–1.94)	1.10 (0.53–2.30)	0.416
Multivariable adjusted model 2	1.00 (Ref.)	0.82 (0.39–1.73)	0.94 (0.45–1.94)	1.11 (0.53–2.31)	0.423
Repeated measurements	1.00 (Ref.)	0.90 (0.43–1.89)	0.98 (0.49–1.98)	1.13 (0.57–2.25)	0.647
	**Low-Fat Dairy Product Consumption**				
	**Q1 (Ref.)**	**Q2**	**Q3**	**Q4**	***p* for trend**
*N*	2493	2493	2493	2492	
Median low-fat dairy product consumption	0.1	1	1.7	3	
Incident cases	29	9	13	16	
Person-years of follow-up	26,719	24,414	23,747	23,052	
Incidence rate/10,000 person-years	10.85	3.68	5.47	6.94	
Age-adjusted HR (95% CI)	1.00 (Ref.)	0.40 (0.19–0.83)	0.49 (0.25–0.95)	0.60 (0.32–1.12)	0.159
Multivariable adjusted model 1	1.00 (Ref.)	0.36 (0.17–0.78)	0.49 (0.23–0.99)	0.61 (0.28–1.33)	0.209
Multivariable adjusted model 2	1.00 (Ref.)	0.36 (0.17–0.78) *	0.49 (0.23–0.99) *	0.61 (0.28–1.33)	0.207
Repeated measurements	1.00 (Ref.)	0.26 (0.11–0.59) **	0.48 (0.25–0.92) *	0.55 (0.28–1.07)	0.198
	**Fermented Dairy Product Consumption**				
	**Q1 (Ref.)**	**Q2**	**Q3**	**Q4**	***p* for trend**
*N*	2493	2493	2493	2492	
Median fermented dairy product	0.6	1	1.7	2.7	
Incident cases	16	13	23	15	
Person-years of follow-up	24,455	25,100	24,333	24,044	
Incidence rate/10,000 person-years	6.54	5.17	9.45	6.23	
Age-adjusted HR (95% CI)	1.00 (Ref.)	0.80 (0.38–1.67)	1.37 (0.72–2.60)	0.90 (0.44–1.82)	0.995
Multivariable adjusted model 1	1.00 (Ref.)	0.86 (0.40–1.88)	1.56 (0.78–3.13)	1.13 (0.51–2.53)	0.555
Multivariable adjusted model 2	1.00 (Ref.)	0.87 (0.41–1.83)	1.56 (0.78–3.13)	1.13 (0.51–2.53)	0.552
Repeated measurements	1.00 (Ref.)	0.88 (0.41–1.86)	1.41 (0.71–2.80)	0.89 (0.43–1.88)	0.970

* *p* < 0.05, ** *p* < 0.01. Ref., reference. Results from Cox regression models. All Cox models were stratified for age (decades) and recruitment period. Model 1 additionally adjusted for height, years at university, family history of breast cancer (none, after 45 years, or before 45 years), smoking status (never smoker, former smoker, current smoker), lifetime tobacco exposure (pack-years), physical activity (METs/week), TV watching (h/day), alcohol intake (g/day, continuous), BMI (<25, 25–30, ≥30), age of menarche (<10 years, 10–11 years, 12–13 years, ≥14 years), history of pregnancy (age <25 years and nulliparous, age ≥25 years and nulliparous, first pregnancy before 25 years, first pregnancy between 25 and 30 years of age, first pregnancy being 30 years old or older), months of breastfeeding (continuous), energy intake (kcal/day), energy-adjusted intake of calcium, vitamin D, and saturated fat from non-dairy products (tertiles), coffee consumption (<1 and ≥1), sugar-sweetened beverage consumption (never/seldom, ≥1 serving per week), and oral contraceptives (yes/no). Model 2 additionally adjusted for Mediterranean diet adherence (score Trichopolou) (continuous). Repeated measurements were adjusted for the same variables as Model 1, using cumulative averages for all dietary variables.

**Table 4:** Hazard ratio (HR) and 95% confidence intervals (CI) of confirmed postmenopausal breast cancer cases according to the categories of baseline total dairy, whole-fat, low-fat, and fermented dairy product consumption among 3299 women of the SUN Project.

	**Total Dairy Product Consumption**				
	**Q1 (Ref.)**	**Q2**	**Q3**	**Q4**	***p* for trend**
*N*	825	825	825	824	
Median dairy product	1.7	2.6	3.6	5	
Incident cases	15	10	7	11	
Person-years of follow-up	5938	5252	5403	5891	
Incidence rate/10,000 person-years	30.31	13.32	12.95	18.67	
Age-adjusted HR (95% CI)	1.00 (Ref.)	0.38 (0.15–0.91)	0.37 (0.15–0.89)	0.58 (0.27–1.24)	0.178
Multivariable adjusted model 1	1.00 (Ref.)	0.37 (0.16–0.86)	0.34 (0.13–0.89)	0.59 (0.20–1.75)	0.484
Multivariable adjusted model 2	1.00 (Ref.)	0.36 (0.15–0.88) *	0.33 (0.13–0.88) *	0.57 (0.19–1.74)	0.462
Repeated measurements	1.00 (Ref.)	0.28 (0.10–0.76) *	0.42 (0.18–0.96) *	0.59 (0.26–1.34)	0.283
	**Whole-Fat Dairy Product Consumption**				
	**Q1 (Ref.)**	**Q2**	**Q3**	**Q4**	***p* for trend**
*N*	825	825	825	824	
Median whole-fat dairy	0.5	1	1.6	2.9	
Incident cases	10	15	9	9	
Person-years of follow-up	7164	5578	5078	4664	
Incidence rate/10,000 person-years	16.74	23.30	19.69	17.15	
Age-adjusted HR (95% CI)	1.00 (Ref.)	1.55 (0.69–3.46)	0.94 (0.38–2.33)	0.98 (0.39–2.44)	0.666
Multivariable adjusted model 1	1.00 (Ref.)	1.62 (0.70–3.75)	1.20 (0.47–3.06)	0.87 (0.31–2.37)	0.394
Multivariable adjusted model 2	1.00 (Ref.)	1.60 (0.69–3.72)	1.18 (0.46–3.02)	0.85 (0.31–2.34)	0.371
Repeated measurements	1.00 (Ref.)	1.49 (0.69–3.21)	1.20 (0.47–3.07)	0.70 (0.27–1.84)	0.338
	**Low-Fat Dairy Product Consumption**				
	**Q1 (Ref.)**	**Q2**	**Q3**	**Q4**	***p* for trend**
*N*	825	825	825	824	
Median low-fat dairy	0.1	1	2	3.4	
Incident cases	13	11	11	8	
Person-years of follow-up	5396	5175	5367	6546	
Incidence rate/10,000 person-years	24.09	21.25	16.76	15.27	
Age-adjusted HR (95% CI)	1.00 (Ref.)	0.85 (0.39–1.88)	0.62 (0.26–1.47)	0.58 (0.24–1.39)	0.186
Multivariable adjusted model 1	1.00 (Ref.)	1.12 (0.48–2.56)	1.01 (0.40–2.53)	1.13 (0.40–3.17)	0.848
Multivariable adjusted model 2	1.00 (Ref.)	1.13 (0.49–2.59)	1.01 (0.40–2.54)	1.12 (0.40–3.15)	0.830
Repeated measurements	1.00 (Ref.)	1.19 (0.51–2.78)	0.60 (0.22–1.66)	0.83 (0.36–1.93)	0.443
	**Fermented Dairy Product Consumption**				
	**Q1 (Ref.)**	**Q2**	**Q3**	**Q4**	***p* for trend**
*N*	825	825	825	824	
Median fermented dairy	0.6	1	1.7	2.8	
Incident cases	12	12	9	10	
Person-years of follow-up	5980	5152	5745	5607	
Incidence rate/10,000 person-years	20.06	23.28	15.66	17.83	
Age-adjusted HR (95% CI)	1.00 (Ref.)	0.95 (0.42–2.12)	0.70 (0.29–1.66)	0.81 (0.34–1.88)	0.555
Multivariable adjusted model 1	1.00 (Ref.)	1.34 (0.38–3.10)	0.96 (0.38–2.44)	1.24 (0.47–3.25)	0.855
Multivariable adjusted model 2	1.00 (Ref.)	1.33 (0.58–3.09)	0.96 (0.38–2.44)	1.24 (0.46–3.26)	0.848
Repeated measurements	1.00 (Ref.)	1.20 (0.53–2.76)	0.64 (0.25–1.62)	1.07 (0.43–2.63)	0.856

* *p* < 0.05. Results from Cox regression models. All Cox models were stratified for age (decades) and recruitment period. Model 1 additionally adjusted for height, years at university, family history of breast cancer (none, after 45 years, or before 45 years), smoking status (never smoker, former smoker, current smoker), lifetime tobacco exposure (pack-years), physical activity (METs/week), TV watching (h/day), alcohol intake (g/day, continuous), BMI (<25, 25–30, ≥30), age of menarche (<10 years, 10–11 years, 12–13 years, ≥14 years), age at menopause (<50 years, ≥50 years), history of pregnancy (age <25 years and nulliparous, age ≥25 years and nulliparous, first pregnancy before 25 years, first pregnancy between 25 and 30 years of age, first pregnancy being 30 years old or older), months of breastfeeding (continuous), use of hormone replacement therapy (yes/no) and its duration (continuous), energy intake (kcal/day), energy-adjusted intake of calcium, vitamin D, and saturated fat from non-dairy products (tertiles), coffee consumption (<1 and ≥1), sugar-sweetened beverage consumption (never/seldom, ≥1 serving per week), and oral contraceptives (yes/no). Model 2 additionally adjusted for Mediterranean diet adherence (score Trichopolou) (continuous). Repeated measurements were adjusted for the same variables as Model 1, using cumulative averages for all dietary variables.

## Data Availability

The data presented in this study are available upon request from the corresponding author.

## Supplementary Materials

The following are available online at https://www.mdpi.com/2072-6643/13/2/687/s1: Table S1. Description of different dairy products considered in the different categories of main food groups as baseline exposures; Table S2. Sensitivity analyses. Hazard ratio (HR) and 95% confidence interval (CI) for overall breast cancer using the Mediterranean Diet Adherence Screener (score MEDAS); Table S3. Sensitivity analyses. Hazard ratio (HR) and 95% confidence interval (CI) for premenopausal breast cancer using the Mediterranean Diet Adherence Screener (score MEDAS); Table S4. Sensitivity analyses. Hazard ratio (HR) and 95% confidence interval (CI) for premenopausal breast cancer using the Mediterranean Diet Adherence Screener (score MEDAS).
