# Supplementary material for: Dairy Consumption and Incidence of Breast Cancer in the ‘Seguimiento Universidad de Navarra’ (SUN) Project
Source: Nutrients. 2021 Feb 21;13(2):687. doi: 10.3390/nu13020687 (PMC7924827; doi:10.3390/nu13020687)
Supplement: Supplementary file 1 [file nutrients-13-00687-s001.pdf]

Supplemental material

Supplemental Table S1. Description of different dairy products considered in the different categories of main food groups as baseline exposures.

Food groups categories	Specific sub-groups
**All dairy products**	Whole- fat milk
	Semi/skimmed milk
	Condensed milk
	Cream or milk cream
	Milkshakes
	Whole yogurt
	Skimmed yogurt
	Petit-suisse
	Cottage cheese or curd
	Portioned or creamy cheese
	Cured and semi-cured cheeses
	Fresh cheese
	Custand, flan, pudding
	Ice-creams
**Whole-fat dairy product consumption**	Whole- fat milk
	Condensed milk
	Cream or milk cream
	Milkshakes
	Whole yogurt/ Petit-suisse
	Portioned or creamy cheese
	Custand, flan, pudding
	Ice-creams
	Cured and semi-cured cheeses
**Low-fat dairy product consumption**	Semi/skimmed milk
	Skimmed yogurt
	Fresh cheese
**Fermented dairy product consumption**	Whole yogurt
	Skimmed yogurt
	Petit-suisse
	Cottage cheese or curd/Fresh cheese
	Portioned or creamy cheese
	Cured and semi-cured cheeses
	Fresh cheese

Supplemental Table S2: Hazard ratio (HR) and 95% confidence intervals (CI) of confirmed breast cancer cases according to the categories of baseline total dairy, whole-fat, low-fat and fermented dairy product consumption among 10,930 women of the SUN Project.

	Dairy product consumption				
	**Q1 (Ref)**	**Q2**	**Q3**	**Q4**	***P for trend***
N	2733	2732	2733	2732	
Median Dairy product	1.6	2.5	3.4	4.8	
Cases	41	23	19	36	
Person-years of follow-up	30,609	30,506	31,368	30,811	
Incidence rate/10,000 person-years	13.39	7.53	6.05	11.68	
Age-adjusted HR (95% CI)	1.00 (Ref.)	0.59 (0.35-0.99)	0.45 (0.26-0.78)	0.89(0.57-1.39)	0.461
Multivariable adjusted model 1	1.00 (Ref.)	0.64 (0.37-1.09)	0.53 (0.28-1.00)	1.19 (0.61-2.31)	0.843
Multivariable adjusted model 2	1.00 (Ref.)	0.62 (0.36-1.07)	0.51 (0.27-0.97)*	1.15 (0.58-2.26)	0.924
	**Whole-fat dairy product consumption**				
	**Q1 (Ref)**	**Q2**	**Q3**	**Q4**	***P for trend***
N	2733	2732	2733	2732	
Median whole-fat dairy product	0.6	1.19	1.18	3.1	
Cases	29	32	28	30	
Person-years of follow-up	29,397	29,672	31,367	32,859	
Incidence rate/10,000 person-years	9.86	10.78	8.92	9.12	
Age-adjusted HR (95% CI)	1.00 (Ref.)	1.19 (0.71-1.97)	1.01 (0.60-1.72)	1.09 (0.65-1.83)	0.889
	**Low-fat dairy product consumption**				
	**Q1 (Ref)**	**Q2**	**Q3**	**Q4**	***P for trend***
N	2,733	2732	2,733	2732	
Median low-fat dairy product	0.67	0.68	1.5	3	
Cases	44	23	24	28	
Person-years of follow-up	33,043	30,357	29,727	29,727	
Incidence rate/10,000 person-years	13.31	7.57	8.07	9.28	
Age-adjusted HR (95% CI)	1.00 (Ref.)	0.57 (0.34-0.95)	0.58 (0.35-0.96)	0.63 (0.39-1.03)	0.070
	**Fermented dairy product consumption**				
	**Q1 (Ref)**	**Q2**	**Q3**	**Q4**	***P for trend***
N	2733	2732	2733	2732	
Median fermented dairy product	0.5	1.04	1.6	2.6	
Cases	30	29	33	27	
Person-years of follow-up	31,229	30,973	30,896	30,196	
Incidence rate/10,000 person-years	9.60	9.36	10.68	8.94	
Age-adjusted HR (95% CI)	1.00 (Ref.)	0.99 (0.59 - 1.65)	1.08 (0.66-1.78)	0.91 (0.54-1.53)	0.835

*p value < 0.05. Results from Cox regression models. All Cox models were stratified for age (decades) and recruitment period. **Model 1** additionally adjusted for height, years at university, family history of BC (none, after 45 years or before 45 years), smoking status (never smoker, former smoker, current smoker), lifetime tobacco exposure (pack-years), physical activity (Mets-h/week), TV-watching (h/day), alcohol intake (g/day, continuous), BMI (<25, 25-<30, ≥30), age of menarche (< 10 years, 10–11 years, 12–13 years, ≥ 14 years), age at menopause (< 50 years, ≥ 50 years), history of pregnancy (age < 25 years & nulliparous, age ≥ 25 years &nulliparous, first pregnancy before 25 years, first pregnancy between 25 & 30 years of age, first pregnancy being 30 years old or older), months of breastfeeding (continuous), use of hormone replacement therapy (yes/no) and its duration (continuous), energy intake(kcal/day), energy-adjusted intake of calcium, vitamin D and saturated fat from non-dairy products (tertiles), coffee consumption (<1, ≥1) , sugar-sweetened beverage consumption (never/seldom, ≥1 serving per week) and oral contraceptives (yes/no). **Model 2** additionally adjusted for Mediterranean Diet Adherence Screener (score MEDAS without alcohol and dairy product items) (continuous).

Supplemental Table S3: Hazard ratio (HR) and 95% confidence intervals (CI) of confirmed premenopausal breast cancer cases according to the categories of baseline total dairy, whole-fat, low-fat and fermented dairy product consumption among 9,971 women of the SUN Project.

	Dairy product consumption				
	**Q1 (Ref)**	**Q2**	**Q3**	**Q4**	***P for trend***
Premenopausal BC					
N	2493	2493	2493	2492	
Median dairy product	1.8	2.7	3.6	4.9	
Incident Cases	20	16	9	22	
Person-years of follow-up	23,954	24,344	25,245	24,389	
Incidence rate/10,000 person-years	8.34	6.57	3.56	9.02	
Age-adjusted HR (95% CI)	1.00 (Ref.)	0.84 (0.43- 1.62)	0.42 (0.19-0.92)	1.08 (0.58-1.98)	0.933
Multivariable adjusted model 1	1.00 (Ref.)	0.88 (0.44-1.77)	0.50 (0.19-1.26)	1.37 (0.59-3.20)	0.447
Multivariable adjusted model 2	1.00 (Ref.)	0.88 (0.43- 1.78)	0.49 (0.19-1.27)	1.36 (0.58-3.22)	0.456
	**Whole-fat dairy product consumption**				
	**Q1 (Ref)**	**Q2**	**Q3**	**Q4**	***P for trend***
Premenopausal BC					
N	2493	2493	2493	2492	
Mean whole-fat dairy product	0.6	1.2	1.8	3	
Incident cases	16	14	17	20	
Person-years of follow-up	21,544	23,534	25,522	27,332	
Incidence rate/10,000 person-years	7.42	5.94	6.66	7.31	
Age-adjusted HR (95% CI)	1.00 (Ref.)	0.76 (0.37-1.57)	0.94 (0.47-1.85)	1.08 (0.56-2.08)	0.603
Multivariable adjusted model 1	1.00 (Ref.)	0.75 (0.36-1.58)	0.95 (0.48-1.88)	1.17 (0.62-2.21)	0.416
Multivariable adjusted model 2	1.00 (Ref.)	0.75 (0.36-1.57)	0.95 (0.48-1.89)	1.17 (0.61-2.22)	0.423
	**Low-fat dairy product consumption**				
	**Q1 (Ref)**	**Q2**	**Q3**	**Q4**	***P for trend***
Premenopausal BC					
N	2493	2493	2493	2492	
Median low-fat dairy product	0.1	1	1.7	3	
Incident cases	29	9	13	16	
Person-years of follow-up	26,719	24,414	23,747	23,052	
Incidence rate/10,000 person-years	10.85	3.68	5.47	6.94	
Age-adjusted HR (95% CI)	1.00 (Ref.)	0.40 (0.19-0.83)	0.49 (0.25-0.95)	0.60 (0.32-1.12)	0.159
Multivariable adjusted model 1	1.00 (Ref.)	0.41 (0.19- 0.85)	0.48 (0.23- 0.99)	0.56 (0.24-1.30)	0.207
Multivariable adjusted model 2	1.00 (Ref.)	0.41 (0.19- 0.86)*	0.48 (0.23-1.00)*	0.56 (0.24-1.30)	0.207
	**Fermented dairy product consumption**				
	**Q1 (Ref)**	**Q2**	**Q3**	**Q4**	***P for trend***
Premenopausal BC					
N	2493	2493	2493	2492	
Median fermented dairy product	0.6	1	1.7	2.7	
Incident cases	16	13	23	15	
Person-years of follow-up	24,455	25,100	24,333	24,044	
Incidence rate/10,000 person-years	6.54	5.17	9.45	6.23	
Age-adjusted HR (95% CI)	1.00 (Ref.)	0.80 (0.38-1.67)	1.37 (0.72-2.60)	0.90 (0.44-1.82)	0.995
Multivariable adjusted model 1	1.00 (Ref.)	0.86 (0.40-1.88)	1.53 (0.78- 2.98)	1.10 (0.54-2.24)	0.555
Multivariable adjusted model 2	1.00 (Ref.)	0.86 (0.39-1.89)	1.53 (0.78-2.98)	1.10 (0.56-2.24)	0.552

*p value < 0.05. Results from Cox regression models. All Cox models were stratified for age (decades) and recruitment period. **Model 1** additionally adjusted for height, years at university, family history of BC (none, after 45 years or before 45 years),smoking status (never smoker, former smoker, current smoker), lifetime tobacco exposure (pack-years), physical activity (Mets/week), hours tv, alcohol intake (g/day,

continuous), BMI (<25, 25-<30, ≥30), age of menarche (< 10 years, 10–11 years, 12–13 years, ≥ 14 years), history of pregnancy (age < 25 years & nulliparous, age ≥ 25 years &nulliparous, first pregnancy before 25 years, first pregnancy between 25 & 30 years of age, first pregnancy being 30 years old or older),months of breastfeeding (continuous), energy intake (kcal/day), energy-adjusted intake of calcium, vitamin D and saturated fat from non-dairy products (tertiles), coffee consumption (<1 and ≥1), sugar-sweetened beverage consumption (never/seldom, ≥1 serving per week) and oral contraceptives (yes/no). **Model 2** additionally adjusted for Mediterranean Diet Adherence Screener (score MEDAS without alcohol and dairy product items) (continuous).

Supplemental Table S4: Hazard ratio (HR) and 95% confidence intervals (CI) of confirmed postmenopausal breast cancer cases according to the categories of baseline total dairy, whole-fat, low-fat and fermented dairy product consumption among 3,299 women of the SUN Project.

.	**Dairy product consumption**				
	**Q1 (Ref)**	**Q2**	**Q3**	**Q4**	***P for trend***
Postmenopausal BC					
N	825	825	825	824	
Median dairy product	1.7	2.6	3.6	5	
Incident Cases	15	10	7	11	
Person-years of follow-up	5,938	5,252	5,403	5,891	
Incidence rate/10,000 person-years	30.31	13.32	12.95	18.67	
Age-adjusted HR (95% CI)	1.00 (Ref.)	0.38 (0.15-0.91)	0.37 (0.15-0.89)	0.58 (0.27-1.24)	0.178
Multivariable adjusted model 1	1.00 (Ref.)	0.37 (0.16-0.86)	0.34 (0.13-0.89)	0.60 (0.20-1.76)	0.484
Multivariable adjusted model 2	1.00 (Ref.)	0.37 (0.15-0.87)*	0.34 (0.13-0.88)*	0.60 (0.21-1.74)	0.486
	**Whole-fat dairy product consumption**				
	**Q1 (Ref)**	**Q2**	**Q3**	**Q4**	***P for trend***
Postmenopausal BC					
N	825	825	825	824	
Median whole-fat dairy	0.5	1	1.6	2.9	
Incident Cases	10	15	9	9	
Person-years of follow-up	7,164	5,578	5,078	4,664	
Incidence rate/10,000 person-years	16.74	23.30	19.69	17.15	
Age-adjusted HR (95% CI)	1.00 (Ref.)	1.55 (0.69-3.46)	0.94 (0.38-2.33)	0.98 (0.39-2.44)	0.666
Multivariable adjusted model 1	1.00 (Ref.)	1.86 (0.78-4.46)	1.08 (0.40-2.95)	0.89 (0.35-2.28)	0.394
Multivariable adjusted model 2	1.00 (Ref.)	1.86 (0.78-4.46)	1.09 (0.40-2.97)	0.89 (0.34-2.31)	0.404
	**Low-fat dairy product consumption**				
	**Q1 (Ref)**	**Q2**	**Q3**	**Q4**	***P for trend***
Postmenopausal BC					
N	825	825	825	824	
Median low-fat dairy	0.1	1	2	3.4	
Incident Cases	13	11	11	8	
Person-years of follow-up	5,396	5,175	5,367	6,546	
Incidence rate/10,000 person-years	24.09	21.25	16.76	15.27	
Age-adjusted HR (95% CI)	1.00 (Ref.)	0.85 (0.39-1.88)	0.62 (0.26-1.47)	0.58 (0.24-1.39)	0.186
Multivariable adjusted model 1	1.00 (Ref.)	1.07 (0.46-2.49)	0.90 (0.36-2.29)	0.95 (0.35-2.59)	0.848
Multivariable adjusted model 2	1.00 (Ref.)	1.07 (0.46-2.48)	0.90 (0.36-2.30)	0.95 (0.35-2.59)	0.853
	**Fermented dairy product consumption**				
	**Q1 (Ref)**	**Q2**	**Q3**	**Q4**	***P for trend***
Postmenopausal BC					
N	825	825	825	824	
Median fermented dairy	0.6	1	1.7	2.8	
Incident Cases	12	12	9	10	
Person-years of follow-up	5,980	5,152	5,745	5,607	
Incidence rate/10,000 person-years	20.06	23.28	15.66	17.83	
Age-adjusted HR (95% CI)	1.00 (Ref.)	0.95 (0.42-2.12)	0.69 (0.29-1.66)	0.81 (0.34-1.88)	0.555
Multivariable adjusted model 1	1.00 (Ref.)	1.16 (0.49-2.77)	0.90 (0.40-2.03)	1.16 (0.46-2.93)	0.855
Multivariable adjusted model 2	1.00 (Ref.)	1.16 (0.48-2.77)	0.90 (0.40-2.03)	1.15 (0.46-2.92)	0.860

*p value < 0.05. Results from Cox regression models. All Cox models were stratified for age (decades) and recruitment period. **Model 1** additionally adjusted for height, years at university, family history of BC (none, after 45 years or before 45 years), smoking status (never smoker, former smoker, current smoker),

lifetime tobacco exposure (pack-years), physical activity (Mets/week), hours tv, alcohol intake (g/day, continuous), BMI (<25, 25-<30, ≥30), age of menarche (< 10 years, 10–11 years, 12–13 years, ≥ 14 years), age at menopause (< 50 years, ≥ 50 years), history of pregnancy (age < 25 years & nulliparous, age ≥ 25 years &nulliparous, first pregnancy before 25 years, first pregnancy between 25 & 30 years of age, first pregnancy being 30 years old or older), months of breastfeeding (continuous), use of hormone replacement therapy (yes/no) and its duration (continuous), energy intake (kcal/day),energy-adjusted intake of calcium, vitamin D and saturated fat from non-dairy products (tertiles), coffee consumption (<1 and ≥1), sugar-sweetened beverage consumption (never/seldom, ≥1 serving per week) and oral contraceptives (yes/no). **Model 2** additionally adjusted for Mediterranean Diet Adherence Screener (score MEDAS without alcohol and dairy product items) (continuous).
